# Neuronal Agrin Promotes Proliferation of Primary Human Myoblasts in an Age-Dependent Manner

**DOI:** 10.3390/ijms231911784

**Published:** 2022-10-04

**Authors:** Katarina Gros, Urška Matkovič, Giulia Parato, Katarina Miš, Elisa Luin, Annalisa Bernareggi, Marina Sciancalepore, Tomaž Marš, Paola Lorenzon, Sergej Pirkmajer

**Affiliations:** 1 Institute of Pathophysiology, Faculty of Medicine, University of Ljubljana, 1000 Ljubljana, Slovenia; 2 Department of Life Sciences, University of Trieste, 34127 Trieste, Italy; 3 The B.R.A.I.N. Centre for Neuroscience, University of Trieste, 34127 Trieste, Italy

**Keywords:** agrin, skeletal muscle regeneration, myoblast proliferation, Lrp4, MuSK

## Abstract

Neuronal agrin, a heparan sulphate proteoglycan secreted by the α-motor neurons, promotes the formation and maintenance of the neuromuscular junction by binding to Lrp4 and activating muscle-specific kinase (MuSK). Neuronal agrin also promotes myogenesis by enhancing differentiation and maturation of myotubes, but its effect on proliferating human myoblasts, which are often considered to be unresponsive to agrin, remains unclear. Using primary human myoblasts, we determined that neuronal agrin induced transient dephosphorylation of ERK1/2, while c-Abl, STAT3, and focal adhesion kinase were unresponsive. Gene silencing of Lrp4 and MuSK markedly reduced the BrdU incorporation, suggesting the functional importance of the Lrp4/MuSK complex for myoblast proliferation. Acute and chronic treatments with neuronal agrin increased the proliferation of human myoblasts in old donors, but they did not affect the proliferation of myoblasts in young donors. The C-terminal fragment of agrin which lacks the Lrp4-binding site and cannot activate MuSK had a similar age-dependent effect, indicating that the age-dependent signalling pathways activated by neuronal agrin involve the Lrp4/MuSK receptor complex as well as an Lrp4/MuSK-independent pathway which remained unknown. Collectively, our results highlight an age-dependent role for neuronal agrin in promoting the proliferation of human myoblasts.

## 1. Introduction

**Figure 9:**
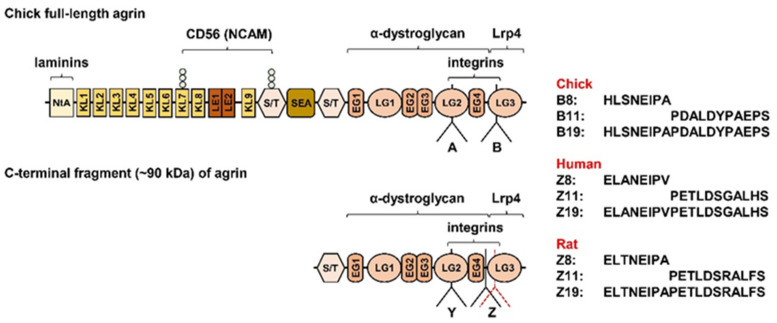
Schematic presentation of agrin structure and some of its major binding partners. Splice sites A and B in chicks correspond to the Y and Z sites, respectively, in mammals. The position of the Z site differs slightly between rat (black line) and human (red dotted line) agrin. Neuronal agrin has an insert with 8 (B8/Z8), 11 (B11/Z11), or 19 (B19/Z19) amino acid residues. The insert with 19 amino acid sequences (B19/Z19) arises from joining of the B8/Z8 and the B11/Z11 sequences. Abbreviations: LE—laminin EGF-like domain; KL—kazal-like domain (aka follistatin-like domain); NtA—N-terminal agrin domain; LG—laminin G-like domain, NCAM—Neural cell adhesion molecule (aka CD56); S/T—serine and threonine-rich domain. Schematic agrin structure is based on structures and sequences published in [3,101] and UniProt: chick agrin (P31696), rat agrin (P25304), and human agrin (O00468).

Neuronal agrin [1,2], a heparan sulphate proteoglycan secreted from the α-motor neurons, promotes formation and maintenance of the neuromuscular junction (NMJ) (reviewed in [3,4,5]) by activating the muscle-specific kinase (MuSK) [6,7,8] via the low-density lipoprotein-related receptor 4 (Lrp4) [9,10] (reviewed in [5,11]). Neuronal agrin binds to Lrp4, the agrin coreceptor, which associates with MuSK and thereby induces its tyrosine kinase activity [9,10,12]. Binding to Lrp4, activation of MuSK, and the NMJ-organizing activity depends on the insert with 8 (Z8), 11 (Z11), or 19 (Z19) amino acid residues at the Z site (B site in chicken) in the C-terminal part of neuronal agrin (aka agrin-Z+) [9,10,13,14] (Figure 9 in Methods). Agrin-Z0, which lacks the critical insert, is therefore incapable of activating Lrp4/MuSK signalling and stabilising the NMJ [9,10,13,14]. Altered degradation of neuronal agrin might contribute to some forms or stages of sarcopenia [15,16,17], a multifactorial syndrome characterised by loss of skeletal muscle function and mass [18,19]. The destabilisation of the NMJ and loss of skeletal muscle mass, which occur due to excessive degradation of agrin in mouse models [20,21,22], are reversed by overexpression or intravenous injection of the cleavage-resistant neuronal agrin [21,23], suggesting that pharmacological therapies that enhance agrin signalling might be effective in some forms or stages of sarcopenia.

As well as promoting the formation and stabilisation of the NMJ, neuronal agrin modulates skeletal muscle regeneration, an essential process that helps to maintain the mass and function of skeletal muscle. The regeneration starts with the activation and proliferation of satellite cells, muscle stem cells which reside between the sarcolemma and basal lamina of mature myofibers [24,25,26]. Satellite cells give rise to myoblasts, which proliferate and then fuse with existing myofibers or with each other to form a multinuclear myotubes [25,26,27]. Once innervated, myotubes develop into mature myofibres, thus replacing the degenerated muscle tissue [27,28]. Neuronal agrin promotes the development of the excitation–contraction (e–c) coupling [29] and sarcolemmal electrical properties [30] of cultured myotubes. In denervated myofibres, neuronal agrin maintains the cytoskeletal architecture [31] and improves the reinnervation [32]. However, overexpression of neuronal agrin in mice tended to reduce the number of immature myofibres via an unknown mechanism [21], indicating that pharmacological activation of agrin signalling might impair skeletal muscle regeneration despite promoting differentiation and innervation of myotubes. 

Proliferation and differentiation are functionally divergent cellular processes that show an inverse relationship, meaning that factors, such as fibroblast growth factor, hepatocyte growth factor (HGF), or Wnt3A protein, which promote myogenic differentiation, often inhibit myoblast proliferation and vice versa [33,34,35,36,37,38]. Untimely or exaggerated stimulation with agrin might therefore promote myoblast differentiation at the expense of their proliferation, thus limiting the formation of new myofibers. However, while neuronal agrin may increase or decrease the proliferation of non-muscle cells [39,40,41,42], its effects on primary human myoblasts, which are thought to be poorly responsive to neuronal agrin such as C2C12 myoblasts [8,10], have not been established. In cultured myotubes neuronal agrin stimulates Lrp4/MuSK, thus leading to activation of c-Abl [43] (reviewed in [44], a non-receptor tyrosine kinase [45], and extracellular signal-regulated kinases 1 and 2 (ERK1/2) [46] (reviewed in [47]), two major mitogen-activated protein kinases [48,49,50]. c-Abl and ERK1/2 are involved in the regulation of proliferation and differentiation of myogenic cells [51,52,53,54] and might represent a potential pathway by which neuronal agrin modulates myoblast function. 

The overall aim of our study was to establish whether and how neuronal agrin affects the function of primary human myoblasts. The specific aims were (1) to establish whether neuronal agrin affects c-Abl and ERK1/2 signalling, (2) to explore whether the Lrp4/MuSK agrin receptor complex modulates proliferation, (3) to determine whether neuronal agrin affects proliferation, and (4) to explore whether activation of Lrp4/MuSK is needed for modulation of proliferation. We found that neuronal agrin transiently suppressed ERK1/2 signalling in primary human myoblasts and that expression of the Lrp4/MuSK complex was important for their proliferation. Neuronal agrin and the C-terminal agrin fragment with or without the Z inserts all increased proliferation of myoblasts of old but not of young donors, which suggests that the effect of neuronal agrin on human myoblast proliferation was age-dependent and at least partially independent of Lrp4/MuSK. Collectively, our results highlight an age-dependent function for neuronal agrin in the modulation of human myoblast proliferation. Furthermore, they exclude a direct negative effect of neuronal agrin on the regenerative potential of myoblasts in humans.

## 2. Results

### 2.1. Detection of c-Abl Signalling Pathway in Primary Human Myoblasts

**Figure 1:**
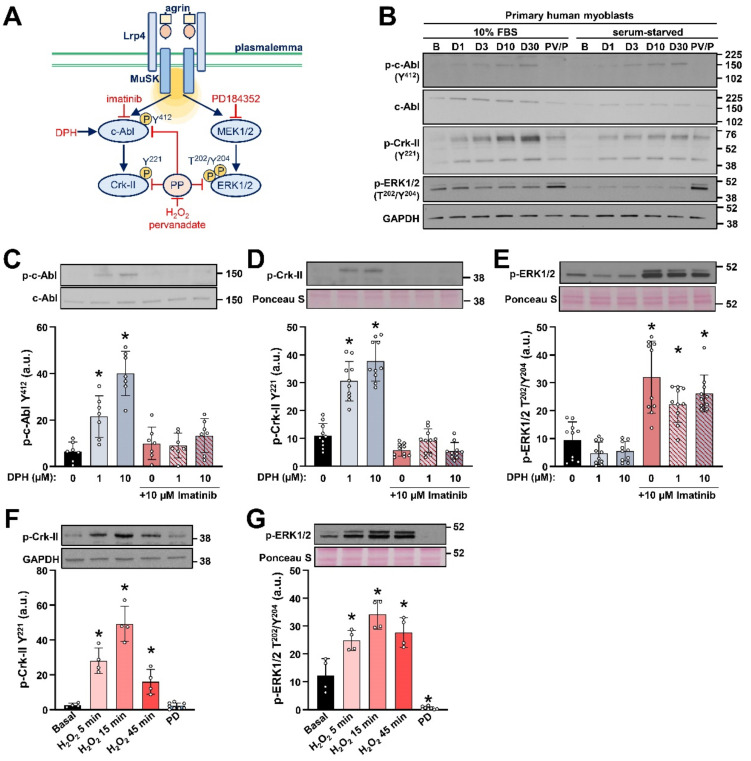
Detection of c-Abl signalling in primary human myoblasts. (**A**) Schematic overview of c-Abl and ERK1/2 signalling from the Lrp4/MuSK receptor complex (based on: [9,10,43,46,63,64]) and the sites of action of DPH, imatinib, PD184352, H_2_O_2_, and pervanadate. P: analysed phosphosites; PP: protein phosphatases. (**B**) Primary human myoblasts (from a 29 year (29Y) old donor) were treated with vehicle (Basal—B) or 1–30 µM DPH (D1-D30) for 4 h or co-treated (PV/P) with 100 µM pervanadate (20 min) and 1 mM H_2_O_2_ (15 min) in absence or presence of serum (10% FBS). Activity of c-Abl and ERK1/2 was estimated by measuring the phosphorylation c-Abl (at Tyr^412^), Crk-II (at Tyr^221^), and ERK1/2 (at Thr^202^/Tyr^204^). Both experiments were performed in duplicate. (**C**–**E**) The phosphorylation of (**C**) c-Abl (at Tyr^412^), (**D**) Crk-II (at Tyr^221^), and (**E**) ERK1/2 (at Thr^202^/Tyr^204^) in primary human myoblasts treated with DPH (1 or 10 µM, 4 h) and/or imatinib (10 µM, during final 30 min) in the serum-free medium. Results are means ± SD, *n* = 10 wells (3 donors: 5Y, 65Y, and 77Y) except for phospho-c-Abl where *n* = 7 wells (2 donors) because in myoblasts of one donor the phosphorylation was below detection level. (**F**,**G**) The phosphorylation of (**F**) Crk-II (at Tyr^221^) and (**G**) ERK1/2 (at Thr^202^/Tyr^204^) in human myoblasts treated with 1 mM H_2_O_2_ (5–45 min) or 10 µM PD184352 (1 h, PD) in the serum-free medium. Results are means ± SD, *n* = 4 wells (2 donors: 6Y and 80Y) except for PD184352 where *n* = 8 (2 donors). * *p* < 0.05 vs. Basal (**B**) or as indicated.

Detection of phosphorylation of endogenous c-Abl proved to be a challenge in different cell models [55,56]. To check if it was possible to measure activating phosphorylation of c-Abl at Tyr^412^ [57] and the phosphorylation of its target Crk-II at Tyr^221^ [58], primary human myoblasts (Figure 1B) and C2C12 myoblasts (Supplementary Figure S1) were treated with 1–30 µM DPH, a selective allosteric activator of c-Abl [56]. In addition, inhibition of protein tyrosine phosphatases with 100 µM pervanadate and 1 mM H_2_O_2_ [59,60,61] was used as an independent strategy to decrease protein dephosphorylation and thereby indirectly increase the c-Abl phosphorylation level [56]. Since the investigation of myoblast proliferation requires the presence of serum, while the signalling pathways are usually assessed in a serum-free media [62], effects of DPH and pervanadate/H_2_O_2_ were assessed both in the presence and absence of 10% foetal bovine serum (FBS).

In primary human myoblasts, DPH induced a concentration-dependent increase in the phosphorylation of Tyr^412^ of c-Abl and Tyr^221^ of Crk-II (Figure 1B). The pervanadate/H_2_O_2_ treatment also increased the phosphorylation of Crk-II, although the increase was less prominent than with the DPH (Figure 1B). The phosphorylation of c-Abl and Crk-II was increased by the DPH and pervanadate/H_2_O_2_ treatment in C2C12 myoblasts (Supplementary Figure S1). In contrast to c-Abl and Crk-II, the phosphorylation of ERK1/2 (Thr^202^/Tyr^204^) was not increased by DPH (Figure 1B and Supplementary Figure S1), indicating that DPH selectively induced the phosphorylation of c-Abl and Crk-II, while the pervanadate/H_2_O_2_ treatment, which inhibits protein tyrosine phosphatases [59,60,61], led to an overall increase in protein phosphorylation.

The primary antibody against the phosphorylated Crk-II (Tyr^221^) showed double DPH-sensitive bands: the first was just above the 38 kDa marker, and the second was between 52 and 76 kDa markers (Figure 1B). For all the quantifications only the lower band, corresponding to the molecular weight of Crk-II (~42 kDa), was considered. To further characterise the responsiveness of this band, c-Abl signalling was tested in more detail by treating them with DPH and imatinib, a selective c-Abl inhibitor [65] (Figure 1C–E). DPH increased the phosphorylation of c-Abl and of Crk-II (Figure 1C,D), which was blocked by the c-Abl inhibitor imatinib, although the low abundance of phospho-c-Abl and phospho-Crk-II, especially in myoblasts of some donors, made for a challenging quantification. DPH tended to suppress the phosphorylation of ERK1/2, while imatinib potently increased it (Figure 1E).

The phosphorylation of Crk-II at Tyr^221^ is induced by reactive oxygen species [66]. As an additional control for the phospho-Crk-II detection, we tested whether the DPH- and imatinib-sensitive band at ~38 kDa responds to H_2_O_2_ in a time-dependent manner. The phosphorylation of ERK1/2 was used as a positive control for the response to H_2_O_2_ [67,68,69]. H_2_O_2_ markedly increased the phosphorylation of Crk-II (Figure 1F) and ERK1/2 (Figure 1G), while the phosphorylation of c-Abl remained below the level of reliable detection. PD184352, a highly selective allosteric inhibitor of MEK1/2 [70], the mitogen activated protein kinase 1 and 2 that phosphorylate and thereby activate ERK1/2 [50], completely suppressed the phosphorylation of ERK1/2 (Figure 1G), but did not alter the phosphorylation of Crk-II (Figure 1F). Collectively, these experiments showed that c-Abl signalling in primary human myoblasts could be followed by measuring the phosphorylation of c-Abl (Tyr^412^) and Crk-II (Tyr^221^), but they also revealed that relatively low intensity of the signal on immunoblots may pose a challenge to their quantification.

### 2.2. AgFL Transiently Suppresses the Phosphorylation of ERK1/2 in Primary Human Myoblasts

**Figure 2:**
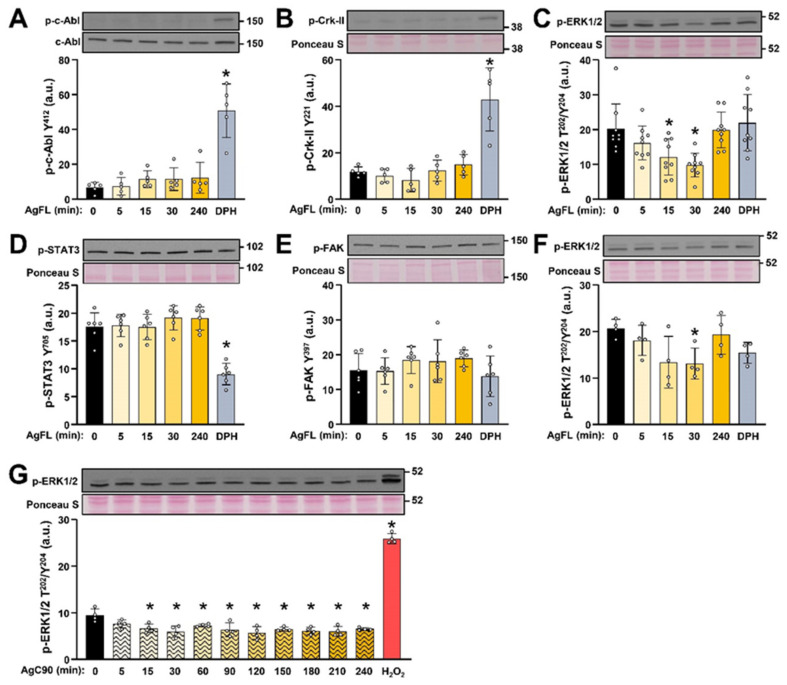
AgFL transiently suppresses the phosphorylation of ERK1/2 in primary human myoblasts. (**A**–**E**) Myoblasts were treated with 1 nM AgFL for 5–240 min and 10 µM DPH for 240 min in the serum-free medium. Activity of c-Abl, ERK1/2, JAK/STAT, and FAK pathways was estimated by measuring the phosphorylation of (**A**) c-Abl (at Tyr^412^), (**B**) Crk-II (Tyr^221^), (**C**) ERK1/2 (Thr^202^/Tyr^204^), (**D**) STAT3 (Tyr^705^), and (**E**) FAK (at Tyr^397^). Results are means ± SD, *n* = 5 donors (7Y, 7Y, 8Y, 15Y, 65Y) except for ERK1/2, where *n* = 9 donors (7Y, 7Y, 8Y, 12Y, 15Y, 17Y, 65Y, 67Y, and 77Y). (**F**) The phosphorylation of ERK1/2 (at Thr^202^/Tyr^204^) in myoblasts treated with 1 nM AgFL for 5–240 min and 10 µM DPH for 240 min in the presence of 10% FBS. Results are means ± SD, *n* = 4 donors (15Y, 17Y, 22Y, and 65 Y). (**G**) The phosphorylation of ERK1/2 (Thr^202^/Tyr^204^) in myoblasts treated with 1 nM AgC90 for 5–240 min or 1 mM H_2_O_2_ for 15 min. Results are means ± SD, *n* = 4 wells (2 donors: 5Y and 80Y). * *p* < 0.05 vs. Basal.

To establish whether c-Abl and ERK1/2 were affected by neuronal agrin, serum-starved myoblasts were treated with 1 nM chick full-length neuronal agrin (AgFL) (Figure 9 in Methods) for up to 4 h, while 10 µM DPH was used as control for c-Abl activation (Figure 2A–E). Serum-free advanced MEM, which contained insulin, transferrin, and albumin, was used to avoid confounding factors in FBS [62].

The phosphorylation of c-Abl (Figure 2A) and Crk-II (Figure 2B) was low under basal conditions as well as during treatment with AgFL, which did not produce any clear effect. Conversely, AgFL induced transient dephosphorylation of ERK1/2 (Figure 2C), which was most prominent at 30 min, but it was completely reversed at 4 h of treatment. Interestingly, AgFL did not alter the phosphorylation of STAT3 (at Tyr^705^) (Figure 2D), a transcription factor that plays an important role in myoblast proliferation [71,72] and differentiation [73,74], neither the phosphorylation of focal adhesion kinase (FAK; at Tyr^397^) (Figure 2E), which participates in the Lrp4-MuSK signalling axis [41,75] and regulation of myoblast proliferation, differentiation, and fusion [76,77,78]. Since myoblasts stop proliferating if the serum is removed, the response of ERK1/2 to AgFL was assessed also in the presence of 10% FBS. The phosphorylation of ERK1/2 was again transiently suppressed at 30 min upon stimulation with 1 nM AgFL (Figure 2F). As an additional control of the AgFL effect on ERK1/2, serum-starved myoblasts were treated with different concentrations of AgFL (0.3–3 nM) for 15 min, which again reduced the phosphorylation of ERK1/2 (Supplementary Figure S2).

To further explore the effect of neuronal agrin on ERK1/2, myoblasts were treated with a 90 kDa C-terminal fragment of rat neuronal agrin (AgC90), which contains the Lrp4 binding site [9,10] but lacks the laminin- and CD56-binding domain(s) (see Figure 9 in Methods). AgC90 rapidly suppressed the phosphorylation of ERK1/2, which did not return to basal during the 4 h treatment (Figure 2G). This result indicated that the C-terminal part of neural agrin was sufficient to induce the dephosphorylation of ERK1/2. Conversely, based on this result, the N-terminal domains of agrin, including those required for binding to laminins and CD56 (see Figure 9 in Methods), seem to be dispensable for this effect even though they could control the kinetics of dephosphorylation.

### 2.3. The Lrp4/MuSK Agrin Receptor Complex Is Required for Proliferation of Primary Human Myoblasts

**Figure 3:**
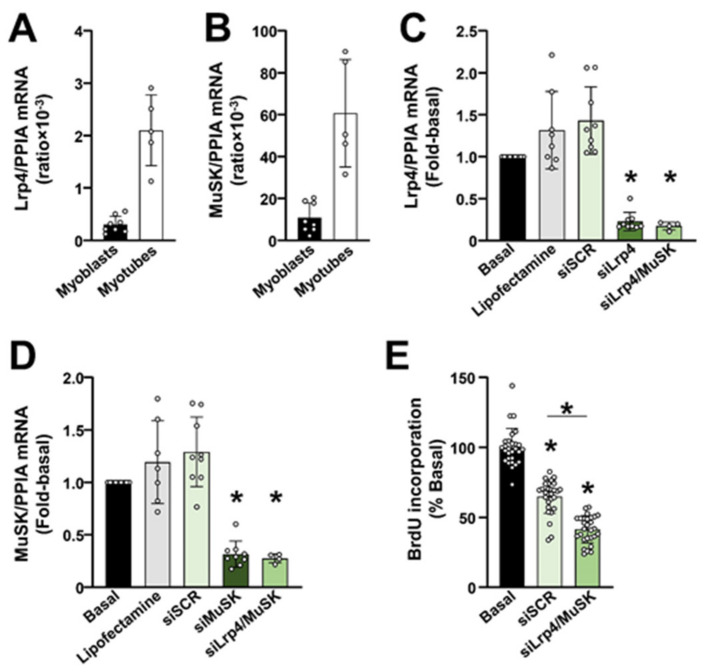
The Lrp4/MuSK agrin receptor complex is required for proliferation of primary human myoblasts. (**A**,**B**) Expression of mRNA for Lrp4 and MuSK in primary human myoblasts was determined using qPCR. Myotubes were used as positive controls. Endogenous control: cyclophilin A (PPIA). Gene expression ratios are reported as means ± SD, *n* = 8 donors for myoblasts and *n* = 5 donors for myotubes. (**C**–**E**) Myoblasts were transfected with scrambled siRNA (siSCR) or siRNAs against Lrp4 and/or MuSK. Untreated (Basal) and Lipofectamine-treated myoblasts were used as additional controls. The mRNA expression of (**C**) Lrp4 and (**D**) MuSK was estimated using qPCR. Endogenous control: PPIA. Gene expression ratios are reported as means ± SD, *n* = 7–9 wells (3 donors: 5Y, 44Y, and 80Y) except for the single knock-down of Lrp4 or MuSK, where *n* = 4 wells (1 donor: 7Y). (**E**) The BrdU incorporation in untreated myoblasts (Basal) and in myoblasts transfected with the scrambled siRNA (siSCR) or a pool of siRNAs against Lrp4 and MuSK (siLrp4/MuSK). Results are means ± SD, *n* = 28 wells (3 donors: 5Y, 44Y, and 80Y). * *p* < 0.05 vs. Basal or as indicated.

Cultured primary human myoblasts expressed Lrp4 (Figure 3A) and MuSK (Figure 3B). To determine whether Lrp4 and MuSK were functionally important for proliferation, myoblasts were transfected with siRNAs, which efficiently suppressed the expression of Lrp4 (Figure 3C) and MuSK (Figure 3D). Gene silencing of Lrp4 and MuSK reduced the incorporation of BrdU by ~37% compared with the scrambled siRNA (siSCR) (Figure 3E). When only Lrp4 was knocked-down the BrdU incorporation was reduced (*p* < 0.05) from 100 ± 19.0% in siSCR-treated myoblasts to 69.3 ± 15.5% (*n* = 16, 1 donor: 7Y). Gene silencing of MuSK alone reduced (*p* < 0.05) the BrdU incorporation from 100 ± 17.6% in siSCR-treated myoblasts to 87.1 ± 10.8% (*n* = 15–16, 1 donor: 7Y). These results indicated a role for the Lrp4/MuSK complex in the proliferation of primary human myoblasts.

### 2.4. Effect of AgFL on Proliferation of Primary Human Myoblasts

**Figure 4:**
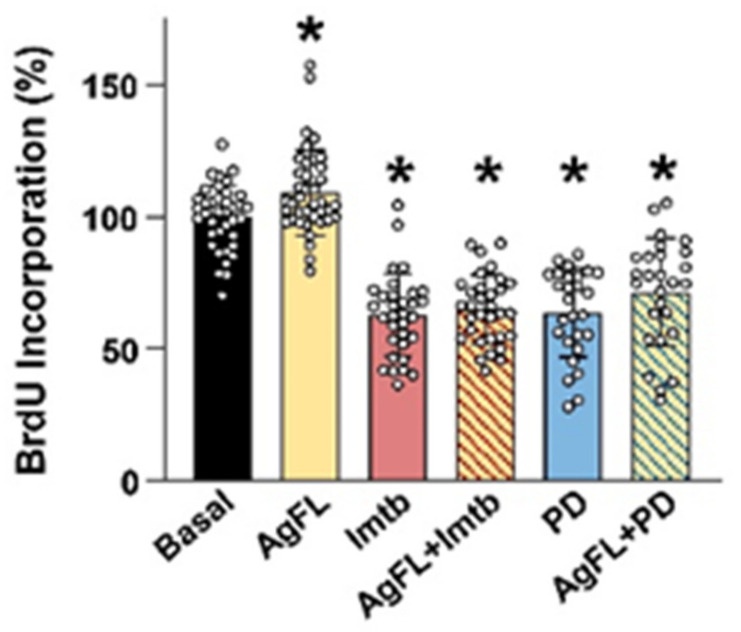
Effect of AgFL on proliferation of primary human myoblasts. Primary human myoblasts were treated with 1 nM AgFL for 24 h in the presence or absence of 10 µM imatinib or 1 µM PD1843521. Myoblast proliferation was estimated by measuring the incorporation of BrdU. Results are means ± SD, *n* = 39 wells (6 donors: 5Y, 7Y, 12Y, 13Y, 60Y and 66Y) for Basal and AgFL, *n* = 32 wells (6 donors: 5Y, 7Y, 13Y, 60Y, 60Y, and 66Y) for imatinib and imatinib + AgFL, *n* = 27 wells (4 donors: 5Y, 7Y, 13Y, and 66Y) for PD1843521 and PD1843521 + AgFL. * *p* < 0.05 vs. Basal.

Inhibition of ERK1/2 leads to differentiation of mouse myoblasts [51], suggesting that the agrin-induced dephosphorylation of ERK1/2 might reduce the proliferation of human myoblasts. Gene silencing of Lrp4/MuSK (Figure 3), which reduced myoblast proliferation, suggested that AgFL may increase proliferation by activating Lrp4/MuSK. Some growth factors, such as insulin growth factor I (IGF-I), may promote myoblast proliferation if ERK1/2 is active or myoblast differentiation if ERK1/2 is inactive [79]. The effect of AgFL on the proliferation of primary myoblasts (Figure 4) was therefore estimated by measuring the BrdU incorporation after a 24 h treatment with 1 nM AgFL in the presence or absence of c-Abl inhibitor imatinib (10 µM) or MEK1/2 inhibitor PD184352 (1 µM). In a set of preliminary experiments in C2C12 myoblasts (Supplementary Figure S3), 1 µM PD184352 completely blocked the basal as well as the imatinib- or the H_2_O_2_-induced phosphorylation of ERK1/2. In human myoblasts, AgFL increased the incorporation of BrdU by ~9%, while imatinib and PD184352 reduced it by ~37% (Figure 4). AgFL did not alter the BrdU incorporation in the presence of imatinib and PD184352, indicating that c-Abl and/or ERK1/2 were required for the effects of AgFL on myoblast proliferation.

### 2.5. Effect of AgFL and AgZ0 on Proliferation of Primary Human Myoblasts Is Age-Dependent

Sorting results for donor age (results in Figure 4), we noticed that an AgFL-stimulated increase in the incorporation of BrdU (127.6 ± 16.7% in the AgFL-treated group vs. 100.0 ± 16.4% in the untreated group, *p* < 0.05) was present only in cultured myoblasts from older donors (60Y and 66Y), while it was absent (102.9 ± 9.8% in the AgFL-treated vs. 100.0 ± 10.5% in the untreated group, *p* > 0.05) in cells of young donors (5Y, 7Y, 12Y, and 13Y). When old myoblasts were exposed to AgFL for 48 h the incorporation of BrdU was again increased from 100.0 ± 13.6% in the untreated myoblasts to 143.5 ± 61.4% (*p* < 0.05) in the AgFL-treated myoblasts (3 donors: 60Y, 66Y, and 77Y, *n* = 26 wells). A similar result was obtained after a 72 h treatment, which increased the BrdU incorporation from 100 ± 41.4% in the untreated group to 164.4 ± 48.3% (*p* < 0.05) in the AgFL-treated group (2 donors: 60Y and 77Y, *n* = 23–24 wells).

**Figure 5:**
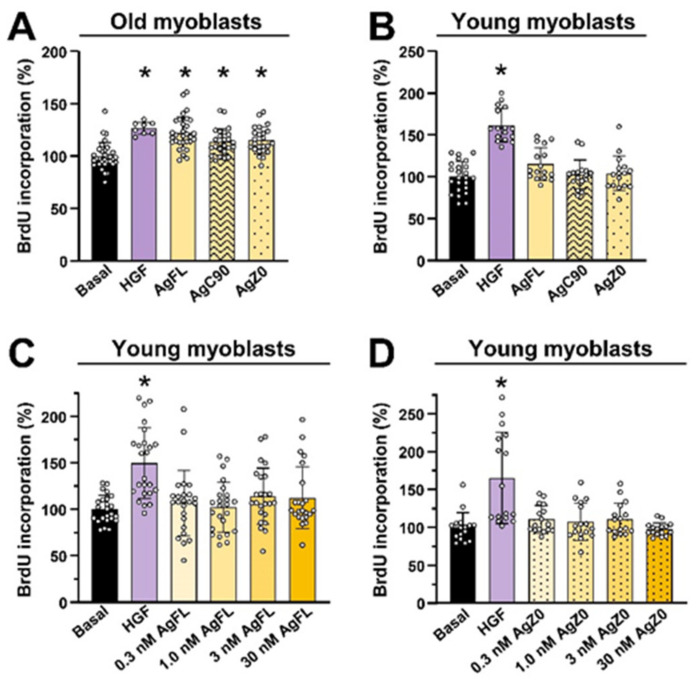
Effect of AgFL and AgZ0 on proliferation of primary human myoblasts is age dependent. Myoblast proliferation was estimated by measuring the incorporation of BrdU and HGF (2.5 nM) was used as a positive control. (**A**) Myoblasts from old donors were treated with 1 nM AgFL or 1 nM AgC90 for 24 h. Results are means ± SD, *n* = 24–32 wells (3 donors: 65Y, 67Y, and 77Y), except for HGF where *n* = 8 wells (1 donor: 77Y). (**B**) Myoblasts from a young donor (12Y) were treated with 1 nM AgFL, AgC90, or AgZ0 for 24 h. Results are means ± SD, *n* = 16 except for Basal, where *n* = 24 wells. (**C**) Myoblasts from young donors were treated with 0.3–30 nM AgFL for 24 h. Results are means ± SD, *n* = 23–24 wells (3 donors: 5Y, 12Y, and 12 Y). (**D**) Myoblasts from young donors were treated with 0.3–30 nM AgZ0 for 24 h. Results are means ± SD, *n* = 16 wells (2 donors: 5Y and 12Y). * *p* < 0.05 vs. Basal.

These results indicated that the effect of AgFL on myoblast proliferation depended on the age of the donor. To verify this apparent age-dependent effect, myoblasts from old (Figure 5A) and young (Figure 5B) donors were treated with 1 nM AgFL, 1 nM AgC90, 1 nM C-terminal fragment of human agrin-Z0 (AgZ0), or 2.5 nM HGF. AgZ0, which lacks the amino acid insert at the Z-site (AgZ0, Figure 9 in Methods) and is, therefore, unable to bind to Lrp4, was used to assess whether activation of Lrp4/MuSK is essential for proliferative effects of neuronal agrin. HGF, a well-characterised mitogen for skeletal muscle cells [27,37,80,81], was used as a positive control.

The BrdU incorporation in old myoblasts was increased by AgFL, AgC90, AgZ0 as well as by HGF (Figure 5A). Conversely, when myoblasts from a young donor were treated with 1 nM AgFL, AgC90, or AgZ0 the BrdU incorporation remained unaltered, although it increased in response to HGF (Figure 5B). In myoblasts of young donors, the incorporation of BrdU remained unaltered even when AgFL (Figure 5C) or AgZ0 (Figure 5D) were used in a range of 0.3–30 nM. On the one hand, these results indicated that the age-dependent effect of AgFL in promoting myoblast proliferation was mediated by Lrp4/MuSK pathway, on the other they unveiled a second signalling mechanism which remains to be investigated.

### 2.6. Long-Term Exposure to AgFL Increases Proliferation of Old Myoblasts

**Figure 6:**
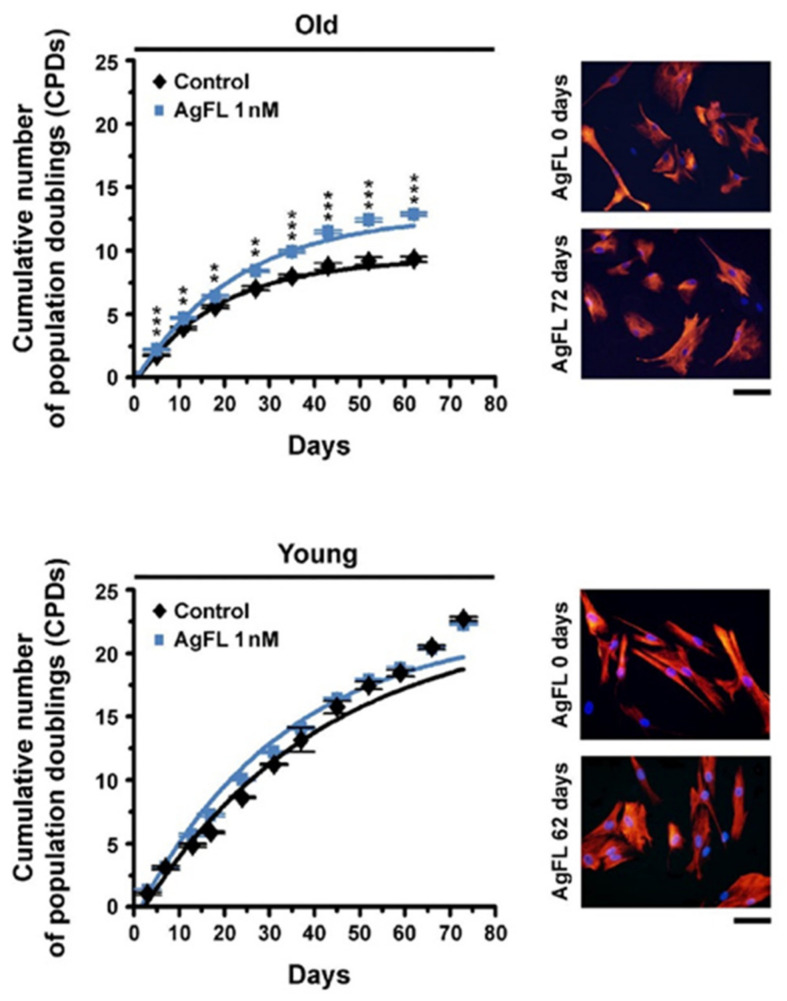
Long-term exposure to AgFL increases proliferation of old myoblasts: primary human myoblasts were grown in the presence of 1 nM AgFL. Representative growth curves for myoblasts from old (top; 58Y) and young (bottom; 7Y) donors are shown (means ± SEM, each experimental point from at least 3 Petri dishes). ** *p* < 0.01, *** *p* < 0.001 vs. Control (Student’s *t*-test). Right, representative optical fields of corresponding cell cultures labelled for desmin at the culture time indicated. Scale bar, 35 µm.

To evaluate the long-term effects of AgFL, old and young myoblasts were grown with or without 1 nM AgFL until they reached the in vitro senescence. Growth curves and the cumulative number of population doublings (CPD) were determined. Treatment with AgFL increased the CPD of old myoblasts already after 2–4 replicative cell cycles and its effect was maintained throughout the in vitro lifespan, including the plateau phase. Overall, CPD was increased by ~15–45% (Table 1 and Figure 6). Conversely, long-term exposure to AgFL did not alter the CPD of young myoblasts (Table 1 and Figure 6), consistent with measurements of the BrdU incorporation. The CPD of young myoblasts remained unaltered even during exposure to lower (100 pM) or higher (50 nM) AgFL concentrations. In old and young cell populations, desmin was determined regularly up until the late passages, when cells approached the cell cycle arrest and ceased to divide (Figure 6). The fraction of desmin-positive cells remained unaltered in the AgFL-treated cells as well as in the control counterparts, indicating that myogenicity was not decreased during AgFL treatment.

### 2.7. Long-Term Exposure to AgFL Does Not Impair Myotube Formation and Establishment of the E–C Coupling Mechanism in Myotubes

**Figure 7:**
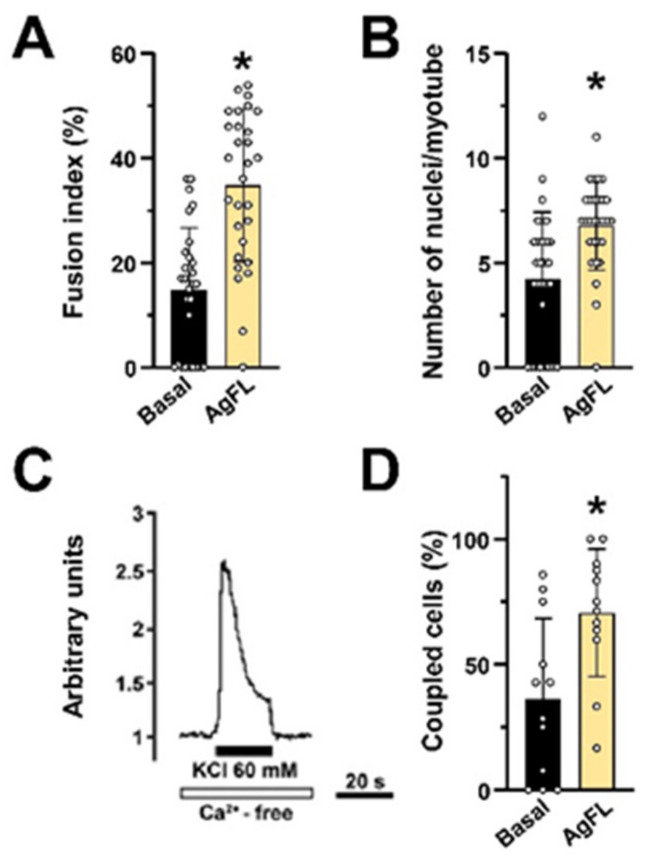
Long-term exposure to AgFL does not impair myotube formation and establishment of the e–c coupling mechanism in myotubes. (**A**) Fusion index (%) and (**B**) the mean number of nuclei per myotube in human myotubes derived from control (fusion index: 14.7 ± 12.0%; nuclei per myotube: 4.2 ± 3.2) and agrin-treated myoblasts (fusion index: 34.8 ± 14.5%; nuclei per myotube: 6.8 ± 2.1) from a 60-year-old donor. Results are means ± SD (*n* = 29 optical fields in A and *n* = 29 myotubes in B). (**C**) A representative Ca^2+^ response to KCl 60 mM in Ca^2+^-free conditions measured in a coupled myotube. (**D**) A histogram showing the percentage of responsive myotubes (coupled cells) from the same donor (control: 36.5 ± 31.7%; AgFL-treated: 70.6 ± 25.3%; *n* = 12 optical fields for both the experimental groups). * *p* < 0.05 vs. Basal.

Sustained proliferation may impair the myogenic differentiation [33,34,35]. We examined whether AgFL-treated myoblasts retained the capability to differentiate despite the enhanced proliferation. The AgFL-treated myoblasts from a 60-year-old donor were induced to differentiate by reducing the serum concentration (see Materials and Methods for details). After 10 days of differentiation, we determined fusion efficiency and the establishment of the skeletal-type e–c coupling mechanism, based on the mechanical link between L-type voltage-operated Ca^2+^ channels and ryanodine receptors [82,83]. Fusion efficiency was evaluated by estimating the fusion index and the number of nuclei per myotube. The fusion index was higher in the AgFL-treated than in the control cells (Figure 7A) as well as the mean number of nuclei per myotube (Figure 7B). The establishment of the e–c coupling mechanism was assessed by Ca^2+^ imaging [84], i.e., measuring the number of cells in which a Ca^2+^ transient was elicited by depolarisation (KCl 60 mM) in Ca^2+^-free conditions (2 mM EGTA excess) (Figure 7C). The percentage of myotubes (± SD) exhibiting a skeletal-type e–c coupling mechanism (Figure 7D) was higher in cultures derived from the AgFL-treated myoblasts than in controls.

## 3. Discussion

The agrin-based pharmacological approaches have been proposed as novel treatments for sarcopenia caused by age-related NMJ degeneration [15,23]. However, overexpression of neuronal agrin was linked to a potentially negative effect on muscle fibre regeneration in mouse models via an unknown mechanism [21]. This result suggested that excessive stimulation with agrin might exacerbate muscle loss and thereby contribute to the sarcopenic phenotype. Here, we report that cultured human primary myoblasts responded to the treatment with neuronal agrin by transient dephosphorylation of ERK1/2, but this was not translated into the suppression of proliferation. Indeed, exposure to neuronal agrin even increased the proliferation of myoblasts obtained from old donors. In addition, we showed that long-term exposure to neuronal agrin did not impair the ability of old myoblasts to differentiate into myotubes with a skeletal-type e–c coupling mechanism. 

**Figure 8:**
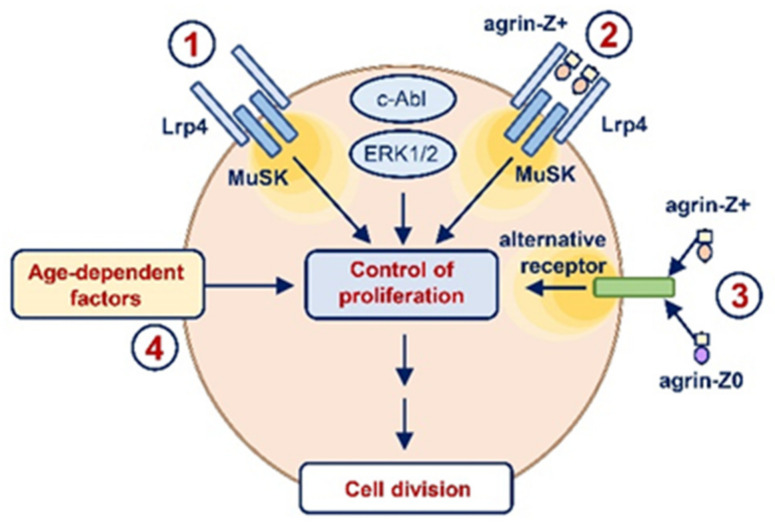
A working model for Lrp4/MuSK receptor complex and neuronal agrin in human myoblasts. Endogenous activity of the Lrp4/MuSK agrin receptor complex is required for proliferation of primary human myoblasts (1). Neuronal agrin (agrin-Z+), which can bind to Lrp4 and activate MuSK, induces transient dephosphorylation of ERK1/2 and increases proliferation of primary human myoblasts in an age-dependent manner (2). Neuronal agrin may affect proliferation also via an alternative agrin receptor (3). Agrin-Z0 exerts its age-dependent effects on myoblast proliferation via the same alternative agrin receptor. Intrinsic defects in geriatric myoblasts (4) likely contribute to the age-specific effect of neuronal agrin on myoblast proliferation.

Our first finding was that gene silencing of Lrp4/MuSK reduced myoblast proliferation in culture. Since the mRNA levels, which we measured, do not necessarily reflect the abundance of the corresponding Lrp4 and MuSK protein, the evidence for the functional role of Lrp4 and MuSK is indirect. Nevertheless, a marked reduction in the Lrp4/MuSK mRNA levels combined with a significant reduction in the BrdU incorporation suggests that Lrp4 and MuSK were not only expressed but were functionally important for primary human myoblasts (Figure 8). These results, therefore, provide a basis for further experiments, such as combining gene silencing with the reintroduction of Lrp4 and/or MuSK, which would enable to validate and further dissect the link between Lrp4/MuSK and proliferation of myoblasts. Lrp4 and MuSK can associate spontaneously, which may lead to activation of MuSK independently of neuronal agrin [10,12]. It is therefore possible that the Lrp4-induced activation of MuSK exerts a positive or at least a permissive effect on the proliferation of primary human myoblasts. Alternatively, deficiency of Lrp4/MuSK due to gene silencing may have disrupted protein–protein interactions that are important for other myoblast functions. In this case, a reduction in proliferative capacity would reflect dysfunction of Lrp4/MuSK-deficient myoblasts rather than a specific role of Lrp4/MuSK in the regulation of cell division. Clearly, these possibilities merit further investigation.

If the “endogenous” activity of the Lrp4/MuSK receptor complex controls the basal proliferation of primary human myoblasts, its activation with exogenous neural agrin enhances the proliferation of myoblasts from old donors (Figure 8). Concerning the possible effect due to an overstimulation of the Lrp4/MuSK receptor complex induced by agrin, our data indicate that even chronic exposure to this neurotrophic factor has no negative impact on the differentiation of old myoblasts. This observation goes against the general rule that proliferation-promoting proteins, such as HGF, suppress myoblast differentiation [37,38], while differentiation-promoting proteins, such as Wnt3A [33], suppress myoblast proliferation [33,36]. Effects of neuronal agrin on myogenesis might therefore be time-dependent, like the effects of IGF-I, which stimulates myoblast proliferation first, but subsequently promotes myogenic differentiation [85,86]. While our study did not address systematically this question, previous data showed that neuronal agrin enhanced the maturation of the e–c coupling mechanism in the in vitro differentiating primary human myoblasts [26]. Moreover, differences in expression of agrin receptors, combinations of receptors, or downstream effectors between myoblasts and myotubes could lead to divergent effects in different stages of myogenesis or skeletal muscle regeneration. 

Investigating the possible signalling cascade activated by neuronal agrin, we observed that AgFL induced transient dephosphorylation of ERK1/2 and increased proliferation of old but not of young myoblasts. On the other hand, both PD184352, which caused sustained dephosphorylation of ERK1/2, and c-Abl inhibitor imatinib, which increased the phosphorylation of ERK1/2, suppressed the proliferation of young and old myoblasts. These results indicate that the mechanisms linking ERK1/2 and control of proliferation differ between primary human skeletal myoblasts and rat smooth muscle cells, in which c-Abl stimulated proliferation by activating ERK1/2 [87]. They also indicate that the relationship between activation of ERK1/2 and proliferation is complex, meaning that the phosphorylation of ERK1/2 does not reliably predict proliferative responses of primary human myoblasts. 

The variable relationship between phosphorylation (activity) of ERK1/2 and proliferation might seem paradoxical, but is not surprising. Indeed, HGF, which stimulates proliferation and inhibits differentiation of myoblasts [37,38], and IGF-I, which stimulates myoblast proliferation as well as differentiation [85], both increase phosphorylation of ERK1/2 [88]. Similarly, ERK1/2 was shown to be important both for the proliferation and differentiation of PC12 cells. However, while epidermal growth factor, which increases proliferation of PC12 cells, activates ERK1/2 only transiently, neural growth factor, which promotes their differentiation, induces a prolonged ERK1/2 activation [89], indicating that different patterns or duration of ERK1/2 activation are specifically and differently decoded by the cells. Importantly, while ERK1/2 is essential for myoblast proliferation, its activation is not sufficient to stimulate myoblast growth [90]. Furthermore, activation of ERK1/2 via the angiopoietin 1/Tie-2 pathway induces the quiescence and reduces proliferation as well as differentiation of cultured myogenic precursor cells [91]. Clearly, ERK1/2 is not a simple on-off switch, whose activation always increases the proliferation of myogenic cells.

Concerning the effect of neuronal agrin on ERK1/2 signalling and its age-dependent effect on myoblast proliferation, considering that the activation (i.e., phosphorylation) of ERK1/2 may lead to quiescence of myogenic precursor cells [91,92], our data indirectly suggest that the agrin-induced dephosphorylation of ERK1/2 might promote re-entry of quiescent myogenic cells into the cell cycle, thus increasing proliferation of human myoblasts. According to this idea neuronal agrin would compensate for a defect that is present in old myoblasts but not in young myoblasts [93,94], which would explain why neuronal agrin increases proliferation in an age-dependent manner (Figure 8). Although the underlying mechanisms remain to be established, alterations in regulatory pathways that control cellular quiescence and cell cycle progression [93,94] could provide an additional explanation for the age-dependent effects of neuronal agrin at the molecular level. Interestingly, while reduced satellite cell availability may contribute to the age-related reduction in the regenerative potential of the skeletal muscle [95], intrinsic defects in geriatric myogenic cells are probably a predominant factor [93,94]. 

One interpretation of the age-dependent effects of neuronal agrin that were observed in our study would therefore be that old myoblasts, which are intrinsically less capable of proliferation, require more support from extrinsic factors, such as neuronal agrin. Importantly, if myoblasts become more dependent on neuronal agrin as skeletal muscle ages, increased degradation of neuronal agrin, which occurs in some forms or stages of sarcopenia [15,16,17], could directly contribute to the reduced regenerative capacity of sarcopenic skeletal muscle. Strategies to enhance the residual function of myogenic progenitors might therefore overcome regenerative limitations of the geriatric skeletal muscle [93,94] and protect more efficiently against muscle loss. In this frame, our data indicate neuronal agrin as a potential novel molecule with which to explore this interesting field.

One of the aims of our study was to establish whether neuronal agrin modulated myoblast function via c-Abl. As assessed by measuring the phosphorylation of c-Abl and Crk-II, AgFL had no effect on c-Abl signalling. However, it needs to be stressed that the abundance of phospho-c-Abl and phospho-Crk-II was low, which means that quantification was more challenging than with phosphoproteins, such as ERK1/2, that are more prominently expressed and/or phosphorylated. The discrepancy between highly abundant total c-Abl and low levels of the phosphorylated c-Abl in unstimulated cells was noted before [55]. Moreover, even DPH-stimulated c-Abl activation may not be detectable with immunoblot unless c-Abl is overexpressed [56]. Therefore, even though our data do not indicate that c-Abl is involved in response to neuronal agrin in primary human myoblasts, its role cannot be confidently excluded.

Another intriguing result of our work is that not only equimolar concentrations of AgC90 (C-terminal agrin-Z+ fragment) but also AgZ0 (C-terminal agrin-Z0 fragment; Figure 9) increased only the proliferation of myoblasts of old donors similarly to the AgFL. In line with our working hypothesis, the mimicking effect of AgC90, which contains the Lrp4 binding site, suggests that the enhancing effect of neuronal agrin on myoblast proliferation is mediated by the activation of the Lrp4/MuSK receptor complex. However, the mimicking effect of AgZ0 indicates that Lrp4/MuSK is not the exclusive receptor responsible for the modulation of myoblast proliferation. Interestingly, both agrin-Z+ and agrin-Z0 isoforms interact with integrins and α-dystroglycan (Figure 9) [96,97,98] and neuronal agrin increases proliferation and attenuates maturation of cardiomyocytes in mouse heart via the disassembly of the α-dystroglycan complex [99]. Therefore, since the α-dystroglycan-binding domain is present in AgFL, AgC90, as well as AgZ0 (Figure 9), it would be important to further examine whether the dephosphorylation of ERK1/2 and/or proliferative effects in skeletal myoblasts from old donors are also mediated by the α-dystroglycan complex.

Our experiments with the C-terminal agrin fragments AgC90 and AgZ0 suggest that laminin as well as CD56 (neural cell adhesion molecule aka NCAM) are dispensable for the effect of neuronal agrin on myoblast proliferation. However, the functional significance of these domains cannot be excluded: kinetics of the dephosphorylation induced by AgFL (transient) were different from the dephosphorylation induced by AgC90, which was more sustained.

In conclusion, although limited by the in vitro approach and by the fact that the mechanism responsible for the age-dependent effect of neuronal agrin partially remains to be identified, our findings put new light on neuronal agrin as a trophic factor enhancing the regenerative potential of aged skeletal muscle. From our data, a complex interaction among different signalling cascades come out in part to be characterised (Figure 8). In line with this, a potential wider range of effects of neuronal agrin emerges and the agrin-based pharmacological approaches proposed to counteract sarcopenia could have benefits not only stabilising the NMJ but also favouring the regenerative capability of myoblasts in the aged muscle.

## 4. Materials and Methods

### 4.1. Reagents and Materials

Cell culture media and reagents were from Invitrogen-Thermo Fisher Scientific (Life Technologies, Paisley, UK) and cell culture materials were from Greiner (Greiner Bio-One GmbH, Germany) unless otherwise specified. Matrigel was from BD Biosciences (Bedford, MA, USA). The BrdU Proliferation Assay Kit was from Calbiochem (KGaA, Darmstadt, Germany). MES running buffer, 4–12% bis-Tris gels were obtained from Bio-Rad (Hercules, CA, USA). Reagents for enhanced chemiluminescence (ECL) and BCA Protein Assay kit were from Thermo Fisher Scientific-Pierce (Rockford, IL, USA). Protein molecular weight marker (full range rainbow marker) was purchased from GE Healthcare (Uppsala, Sweden). PVDF membrane was from Merck-Millipore (Billerica, MA, USA). Agfa X-Ray films (Agfa, Zagreb, Croatia) were developed using Curix 60 developing machine (AGFA HealthCare, Greenville, SC, USA). RNeasy Mini Plus kit was from Qiagen (Hilden, Germany). Real-time PCR was performed using TaqMan chemistry from Applied Biosystems (Thermo Fisher Scientific Inc., Foster City, CA, USA). DPH [i.e., 5-(1,3-diaryl-1H-pyrazol-4-yl) hydantoin, 5-[3-(4-fluorophenyl)-1-phenyl-1H-pyrazol-4-yl]-2,4-imidazolidinedione] and PD1843521 were from Sigma-Aldrich (St. Louis, MO, USA). Imatinib (STI571; Gleevec^®^) was kindly provided by Novartis, Varese, Italy. Unless specified, all other reagents were of analytical grade and from Sigma-Aldrich.

### 4.2. Antibodies

Primary antibodies for immunoblotting were from Cell Signaling Technology (Beverly, MA, USA) (Table 2). Anti-rabbit horseradish peroxidase (HRP)-conjugated secondary antibodies were from Bio-Rad (Hercules, CA, USA). Goat anti-desmin antibody, used for cytochemistry, was from DakoCytomation (Glostrup, Denmark). Rhodamine-conjugated secondary antibody was from Jackson ImmunoResearch Laboratories Inc. (West Grove, PA, USA).

### 4.3. Agrin

Recombinant full-length chick neuronal agrin (cAgrin_7A4B8_) (Figure 9) was purified from the conditioned media of stably transfected HEK-293 cells (kindly provided by G. Fumagalli, University of Verona, Verona, Italy) using mono Q-Sepharose fast flow beads (Amersham Pharmacia Biotech, Piscataway, NJ, USA), as described [29,100]. The 90 kDa recombinant C-terminal fragment of neuronal rat agrin (agrin-Z+, 550-AG), which contains 9 amino acids at the Z-site, and the 83.5 kDa C-terminal fragment of human agrin (6624-AG), which corresponds to the Z- (Z0) agrin, was purchased from R&D Systems (Minneapolis, MN, USA).

### 4.4. Isolation and Purification of Human Skeletal Myoblasts

All procedures were approved by the Ethics Commission at the Ministry of Health of the Republic of Slovenia or by the Ethics Commission of the Medical Faculty of the University of Bonn. Primary myoblast cultures were prepared from skeletal muscle tissue discarded during routine orthopaedic operations. The informed consent was signed by donors or their legal representatives. Muscle samples were collected from donors 5–77 years of age. Human skeletal muscle cell cultures were prepared as described [29,102,103,104]. Briefly, skeletal muscle tissue was cleaned of visible connective and adipose tissue and then cut into small (0.5–1 mm) pieces under the microscope. This was followed by trypsinisation (0.15%; 37 °C for 30–45 min) in Earle’s Balanced Salt Solution (EBSS) to digest the basal lamina and release the satellite cells. The cell suspension was centrifuged (1000 rpm; 5 min), the pellet was re-suspended, and cells were plated into cell culture dishes. Primary cell cultures were propagated at 37 °C in a humidified atmosphere (95% air/5% CO_2_). Advanced MEM, supplemented with 10% (*v*/*v*) FBS, 0.3% (*v*/*v*) Fungizone and 0.15% (*v*/*v*) gentamicin was used as the growth medium (GM). Before reaching confluence, primary cultures were purified using MACS CD56 microbeads (Miltenyi Biotec, Bergisch Gladbach, Germany) in order to separate the myoblasts from other cell types contaminating the primary culture [103]. Only cell cultures with more than 75% of desmin-positive cells (see below) were used for the experiments described here.

### 4.5. Desmin Staining

Desmin is an intermediate filament, which is widely used as a marker for cells of myogenic lineage [105,106]. To determine desmin expression, human skeletal muscle cells were plated onto Matrigel-coated glass coverslips. Cells were subsequently fixated in 4% (*w*/*v*) paraformaldehyde, permeabilised with 0.5% Triton X-100 (5 min at RT) and incubated with primary anti-desmin antibody (1:50 in PBS; overnight at 4 °C) and with rhodamine-conjugated secondary antibody (1:200 in PBS; 1 h at RT). Cell nuclei were stained with Hoechst-33258 (1 µg/mL). Images were taken with a fluorescent microscope Olympus IX81 (Olympus Corporation Tokyo, Japan) at 590 nm for desmin and at 461 nm for Hoechst-stained nuclei. The percentage of myogenic cells was determined by dividing the number of desmin-positive cells by the number of Hoechst-stained nuclei. At least 3 different coverslips were determined.

### 4.6. C2C12 Cell Culture

C2C12 cells (Developmental Studies Hybridoma Bank, Tacoma, Washington) were grown in Dulbecco’s modified Eagle’s medium (DMEM), supplemented with 10% (*v*/*v*) FBS, 0.3% (*v*/*v*) Fungizone and 100 IU/mL penicillin and 100 µg/mL streptomycin at 37 °C in humidified atmosphere (95% air/5% CO_2_).

### 4.7. Pervanadate Treatment

Pervanadate was prepared with a modification of published protocols [60,107]. Briefly, a 100× stock solution containing 10 mM orthovanadate and 16 mM H_2_O_2_ was prepared immediately before the experiment by mixing 934 µL H_2_O with 50 µL of 200 mM sodium orthovanadate and 16 µL of 3% H_2_O_2_, which was prepared freshly from the 30% (*w*/*v*) H_2_O_2_ stock. No attempt was made to remove excess H_2_O_2_ with catalase [60,107] and stock solution was used immediately for cell treatments.

### 4.8. Growth Curves and Cumulative Number of Population Doublings

To assess the long-term effects of agrin on myoblast proliferation, we determined growth curves and the cumulative number of population doublings (CPD) over the entire course of their in vitro lifespan. Myoblasts were plated at the same density (14 cells/mm^2^) and were grown in GM (Ham’s F10 supplemented with FBS (20%; Euroclone, Pero, Italy), 1 mM L-glutamine, 100 units/mL penicillin and 100 µg/mL streptomycin) in the presence or absence of 1 nM AgFL. GM was changed every 3 days. When myoblasts became subconfluent, they were trypsinised and counted using a Coulter counter. At each passage, the mean population doubling (MPD) was calculated as MPD = Log*N*/Log2, where *N* is the number of cells harvested divided by the number of cells seeded. For each experimental point, at least 3 different dishes were analysed. Cell number in each sample was determined in duplicate. Cell cultures were considered to be at the end of their in vitro lifespan (senescent), when their number remained constant during two consecutive sub-cultures. Desmin expression was routinely assessed by immunocytochemistry.

### 4.9. BrdU Incorporation

A bromodeoxyuridine (BrdU) Cell Proliferation Assay Kit was used to estimate the rate of myoblast proliferation. Human myoblasts were plated in 96-well plates (500–2000 cells per well) and were grown in Advanced MEM supplemented with 10% FBS. The day after seeding, myoblasts were exposed to agrin and/or other reagents as described in the Results. Myoblasts were then incubated with BrdU for an additional 16 h. Samples were prepared for BrdU absorbance measurement according to the manufacturer’s protocol. Absorbance was measured at dual wavelengths (450 and 550 nm) using Victor 3 plate reader (PerkinElmer, Shelton, CT, USA). Results are expressed as percentage of the basal. Experiments were performed in at least 4–8 replicates for each treatment per donor.

### 4.10. Quantitative PCR

Total RNA was extracted using the RNeasy Mini Plus Kit (Qiagen, Hilden, Germany) and reverse transcribed with a High-Capacity cDNA Reverse Transcription Kit (Thermo Fisher Scientific-Applied Biosystems). Quantitative PCR (qPCR) was performed on an ABI PRISM SDS 7500, using TaqMan chemistry in a 96-well format (both from Thermo Fisher Scientific-Applied Biosystems). MuSK and Lrp4 mRNA were determined using specific Gene Expression Assays Hs00985763_m1 and Hs00391006_m1, respectively. Cyclophilin A (PPIA, 4333763F) was used as the endogenous control. Gene expression ratios are reported [108], as described [104].

### 4.11. Western Blot

Myoblasts were washed with ice-cold PBS and then harvested with Laemmli buffer 62.5 mM Tris-HCl (pH 6.8), 2% (*w*/*v*) SDS, 10% (*w*/*v*) glycerol, 0.002% (*w*/*v*) bromophenol blue, 5% (*v*/*v*) β-mercaptoethanol) or with lysis buffer (1% (*v*/*v*) Protease Inhibitor Cocktail, 137 mM NaCl, 2.7 mM KCl, 1 mM MgCl_2_, 0.5 mM Na_3_VO_4_, 1% (*v*/*v*) Triton X-100, 1% (*v*/*v*) glycerol, 20 mM Tris, 10 mM NaF, 1 mM EDTA, and 1 mM phenylmethanesulfonyl fluoride). Lysates prepared in lysis buffer were centrifuged at 4 °C (12000 *g*, 15 min) and supernatants were collected. Total protein concentration was measured with a Bicinchoninic Acid (BCA) Protein Assay Kit. Subsequently, samples were diluted to the same final concentration in Laemmli buffer. Protein samples were separated by SDS-PAGE on 4–12% Bis-Tris gel and transferred to a PVDF membrane using Criterion system (Bio-Rad, Hercules, CA, USA). To assess sample loading and the efficiency of the transfer, membranes were stained with 0.1% (*w*/*v*) Ponceau S in 5% (*v*/*v*) acetic acid, as described [62,109,110]. 

Membranes were blocked in 7.5% (*w*/*v*) non-fat milk in TBS-T (10 mM Tris, 137 mM NaCl, 0.02% (*v*/*v*) Tween-20, pH 7.6) and subsequently probed with primary antibodies against proteins of interest and GAPDH (Table 2), which was used as a supplementary loading control, overnight at 4 °C. Following overnight incubation, membranes were incubated with the appropriate HRP-conjugated secondary antibody. Immunoreactive proteins were visualised using enhanced chemiluminescence and analysed with Quantity-One 1-D Analysis Software (Bio-Rad, Hercules, CA, USA).

### 4.12. Myoblast Fusion Efficiency

To estimate the efficiency of myotube formation, the fusion index and the mean number of nuclei per myotube were determined. To trigger differentiation and fusion into myotubes, myoblasts were plated on Matrigel-coated coverslips at 150 cells/mm^2^ in GM. Three days later GM was substituted with differentiation medium (DM): DMEM supplemented with 2% FBS, 100 µg/mL apo-transferrin, 10 µg/mL insulin, 4 mM L-glutamine, 100 units/mL penicillin and 100 µg/mL streptomycin). Fusion index and the mean number of nuclei/myotube were quantified on the 10th day of differentiation. These parameters were determined in randomly chosen optical fields in at least 6 coverslips. Fusion index is expressed as the number of nuclei in myotubes divided by the total number of nuclei. Myotubes were defined as cells having more than 3 nuclei.

### 4.13. Ca^2+^ Imaging in Cultured Myotubes

Ca^2+^ imaging experiments were carried out to check the presence of the skeletal-type excitation–contraction coupling mechanism, assessed as the cell capability to generate an intracellular Ca^2+^ transient when depolarised (KCl 60 mM) in Ca^2+^-free solution (NaCl 140 mM, KCl 2.8 mM, EGTA 2 mM, MgCl_2_ 5 mM, glucose 10 mM, Hepes 10 mM; pH 7.3). To this purpose, cells were seeded and differentiated (7–12 days) on glass coverslips coated with Matrigel. For Ca^2+^ measurements, myotubes were incubated for 30–40 min (at RT) in NES (NaCl 140 mM, KCl 2.8 mM, CaCl_2_ 2 mM, MgCl_2_ 2 mM, glucose 10 mM, Hepes 10 mM; pH 7.3) supplemented with 0.5 mg/mL BSA and the Ca^2+^ indicator fura-2 pentacetoxymethylester (fura-2 AM; 5 µM). The experiments were performed at 37 °C in a temperature-controlled microincubator chamber (Medical System Corporation, Greenvale, NY, USA). Cells were excited alternately at 340 and 380 nm, selected by a monochromator (PolychromeII, TillPhotonics GmbH, Martinsried, Germany). Fluorescence signals were collected by a CCD camera (SensiCam; PCO Computer Optics, Kelheim, Germany) at the acquisition rate of 1 ratio image/s. The monochromator and CCD camera were controlled by TillVision software (TillPhotonics), also used for image processing. The ratio images (340/380) and the corresponding temporal plots (i.e., variations in the mean value of the fluorescence intensity obtained from regions of interest) were calculated offline. In the temporal plots, the fluorescence at rest was assumed to be 1, and only fluorescence variations corresponding to a peak equal to or higher than 10% were considered as a cell response. For each experimental point, at least 6 coverslips were analysed.

### 4.14. Gene Silencing of Lrp4 and MuSK

Human myoblasts were cultured overnight in Advanced MEM with 10% (*v*/*v*) FBS and without antibiotics and antimycotics, reaching 30–50% confluent growth. The transfections were performed using lipofectamine 2000 reagent (Thermo Fisher Scientific) according to the manufacturer’s protocol. Lipofectamine and siRNA were separately diluted in Opti-MEM (Thermo Fisher Scientific) and combined just before adding to the cell culture medium. Final concentrations of siRNA in the medium were: 5 nM against Lrp4 (ID s8287) and 5 nM against MUSK (ID s9084), both Silencer Select siRNA from Ambion—Thermo Fisher Scientifics. Scrambled siRNA (ON-TARGETplus Non-targeting Pool, D-001810-10-20, Dharmacon Horizon Discovery) was used as a negative control. Final concentration of lipofectamine was 2‰ (*v*/*v*). After 24 h incubation in the presence of siRNA, the cell medium was replaced with the complete growth medium. After an additional 24 h the levels of LRP4 and MUSK mRNA were measured with qPCR and 24 h later (72 h after starting lipofection) the proliferation rate was determined by BrdU incorporation.

### 4.15. Statistical Analysis

Statistical analysis was performed using GraphPad Prism 4.0 Software. ANOVA, followed by Bonferroni’s or Dunnett’s *post hoc* test was used to evaluate the differences among several groups. Student *t*-test or Mann–Whitney test was used when only two groups were compared. Results are expressed as means *±* SD or SEM as indicated. Statistical significance was established at *p* < 0.05.

## 5. Conclusions

1. Neuronal agrin induced transient dephosphorylation of ERK1/2 in primary human myoblasts.
2. Lrp4/MuSK was required for proliferation of primary human myoblasts.
3. Acute and chronic treatment with exogenous neuronal agrin increased the proliferation of primary human myoblasts of old donors but did not affect the proliferation of myoblasts of young donors.
4. Stimulation of proliferation by neuronal agrin likely involved Lrp4/MuSK-dependent as well as Lrp4/MuSK-independent pathways.
5. Collectively, our results highlight an age-dependent function for neuronal agrin in the modulation of human myoblast proliferation. Furthermore, they exclude a direct negative effect of neuronal agrin on the regenerative potential of myoblasts in humans.

## Figures and Tables

**Table 1:** Age-dependent effect on AgFL on cumulative number of population doublings. Results are means ± SEM, *n* = number of Petri dishes, ** *p* < 0.01; *** *p* < 0.001 (Student’s *t*-test).

	Cumulative Number of Population Doublings	
Age of Donor (Years)	Control	1 nM AgFL
66	1.63 ± 0.02 *(n* = 5)	2.38 ± 0.09 *** *(n* = 5)
60	4.24 ± 0.09 *(n* = 4)	4.83 ± 0.08 ** *(n* = 4)
58	9.95 ± 0.32 *(n* = 4)	13.07 ± 0.14 *** *(n* = 4)
7	22.70 ± 0.15 *(n* = 3)	22.28 ± 0.08 *(n* = 3)
5	12.21 ± 0.09 *(n* = 4)	12.33 ± 0.07 *(n* = 4)

**Table 2:** Overview of primary and secondary antibodies. Phosphorylation sites Thr^202^ and Tyr^204^ of ERK1 (p44 MAPK) correspond to Thr^185^ and Tyr^187^ of ERK2 (p42 MAPK). Abbreviations: CST—Cell Signalling Technology, GAM—goat-anti-mouse (tetramethylrhodamine isothiocyanate (TRITC)-conjugated antibody (ab6786) or Alexafluor 546-conjugated (A11030)), GAR—goat-anti-rabbit.

Primary Antibody				Secondary Antibody	
Target	Catalogue No.	Dilution	Source	Source/Target	Dilution
c-Abl	CTS #2862	1:1000	rabbit	GAR	1:25,000
p-c-Abl (Tyr^412^)	CST #2865	1:1000	rabbit	GAR	1:8000–1:25,000
p-Crk-II (Tyr^221^)	CST #3491	1:1000	rabbit	GAR	1:12,500–1:25,000
desmin	Dako #M076029	1:50	mouse	GAM	1:200
p-ERK1/2 (Thr^202^/Tyr^204^)	CST #4370	1:20,000	rabbit	GAR	1:25,000–1:50,000
total ERK1/2	CST #4695	1:1000	rabbit	GAR	1:25,000
p-FAK (Tyr^397^)	CST #8556	1:1000	rabbit	GAR	1:8000–1:12,500
GAPDH	CST #5174	1:1000	rabbit	GAR	1:25,000–1:50,000
p-STAT3 (Tyr^705^)	CST #9145	1:2000	rabbit	GAR	1:4000–1:25,000

## Data Availability

Original blots are provided as Supplementary Material. All other data are available from S.P. and P.L. upon reasonable request.

## Supplementary Materials

The following supporting information can be downloaded at: https://www.mdpi.com/article/10.3390/ijms231911784/s1.
