# Supplementary material for: Neuronal Agrin Promotes Proliferation of Primary Human Myoblasts in an Age-Dependent Manner
Source: Int J Mol Sci. 2022 Oct 4;23(19):11784. doi: 10.3390/ijms231911784 (PMC9570459; doi:10.3390/ijms231911784)
Supplement: Supplementary file 1 [file ijms-23-11784-s001.zip › ijms-1886805-supplementary.pdf]

International Journal of *Molecular Sciences*

MDPI

Supplementary Materials: Neuronal Agrin Promotes Proliferation of Primary Human Myoblasts in an Age-Dependent Manner

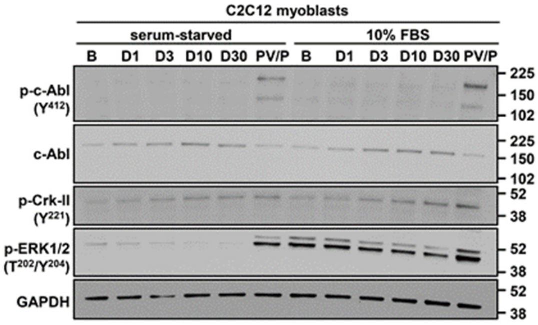

Figure S1. C2C12 myoblasts were treated with 1–30 μM DPH (D1-D30) for 4 h or 100 μM pervanadate for 20 min and 1 mM H_2O_2 for 15 min (PV/P) in the absence or in the presence of serum (10% FBS). The activity of c-Abl and ERK1/2 was estimated by measuring the phosphorylation of Tyr^{412} of c-Abl, Tyr^{221} of Crk-II, and Thr^{202}/Tyr^{204} of ERK1/2. Experiment was performed in duplicate.

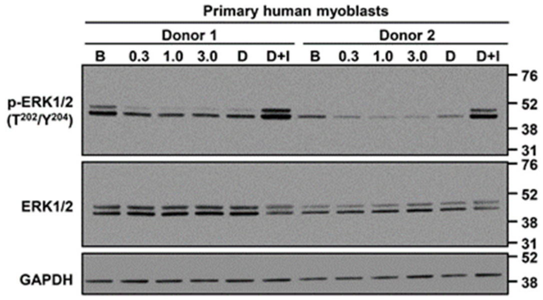

Figure S2. Primary human myoblasts from two donors (12Y and 17Y) were treated with vehicle (Basal, B), AgFL (0.3, 1.0, or 3.0 nM for 15 min), DPH (D), or DPH and Imatinib (D+I). DPH (10 μM) treatment was 4 h and imatinib treatment (10 μM) was 4.5 h. The activity of ERK1/2 was estimated by measuring its phosphorylation at Thr^{202}/Tyr^{204}. Total ERK1/2 and GAPDH are also shown.

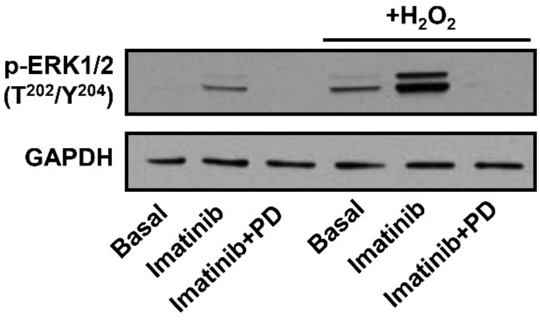

Figure S3. The phosphorylation of ERK1/2 (Thr^{202}/Tyr^{204}) in serum-starved C2C12 myoblasts treated with PD1843521 (PD, 1 μM, 105 min), imatinib (10 μM, 60 min), and/or H_2O_2 (2 mM, 15 min) ($n = 4$ plates). Imatinib was added 15 min after PD1843521, while H_2O_2 was added for the last 15 min of the experiment.
